# *Bogidiellapingxiangensis*, a new species of subterranean Amphipoda from southern China (Bogidiellidae)

**DOI:** 10.3897/zookeys.790.28671

**Published:** 2018-10-15

**Authors:** Yami Zheng, Zhonge Hou, Shuqiang Li

**Affiliations:** 1 Key Laboratory of Zoological Systematics and Evolution, Institute of Zoology, Chinese Academy of Sciences, Beijing 100101, China Institute of Zoology, Chinese Academy of Sciences Beijing China; 2 University of Chinese Academy of Sciences, Beijing 100049, China University of Chinese Academy of Sciences Beijing China

**Keywords:** Amphipod, barcode, cave, China, new species, taxonomy

## Abstract

A new species of subterranean amphipod, *Bogidiellapingxiangensis* Hou & Li, **sp. n.**, is described from Xiongshizilong Cave in Pingxiang City, China. The new species is characterized by having the bases of pereopods III and V expanded; the inner ramus of pleopods I–III with one segment; the telson longer than wide and with the apical margin with a shallow U-shaped excavation. DNA barcode of the new species is documented as support of molecular differences between related species.

## Introduction

The genus *Bogidiella* Hertzog, 1933 contains more than 60 species that are widely distributed in subterranean freshwaters or marine interstitial habitats (Koenemann and Holsinger 1999, Coleman 2009). The genus exhibits typical subterranean adaptive morphology in the loss of eyes and pigmentation, elongated pereopods, and reduced pleopods (Holsinger et al. 2006).

In China, only one species *Bogidiellasinica* Karaman & Sket, 1999 is known. It occurs in the lower storey of the cave system Qixinyan at Guilin, Guangxi Zhuang Autonomous Region. We have tried to get fresh specimens for *B.sinica*, but failed because of tourism in the locality. During a field survey of subterranean amphipods in southern China, a second new species of *Bogidiella* was found in a cave in Jiangxi Province, which is ca. 500 km away from the type locality of *B.sinica*. In this paper, *Bogidiellapingxiangensis* sp. n. is described and illustrated. The barcode sequence of the new species is presented and genetic distances between the new species and known species are calculated to confirm the species delimitation.

## Materials and methods

### Morphological observation

The specimens were collected by sweeping rotten wood with a fine-meshed hand net. Samples were preserved in 95% ethanol in the field, then deposited at -20 °C refrigerator for long-term preservation. The body length of the amphipod was recorded by holding the specimen straight and measuring the distance along the dorsal side of the body from the base of the first antenna to the base of the telson. All dissected appendages were mounted on slides according to the methods described by Holsinger (1967). Appendages were drawn using a Leica DM2500 compound microscope equipped with a drawing tube. Terminology and taxonomic descriptions follow the literature (Leijs et al. 2011), specially the terms ‘‘spines’’ and ‘‘setae’’ are used to distinguish between thin or fine and more robust setal structures. All types and other material are lodged in the Institute of Zoology, Chinese Academy of Sciences (IZCAS), Beijing.

### Molecular methods

A partial fragment of the mitochondrial cytochrome oxidase subunite I (COI) was proposed as a crustacean barcode (Costa et al. 2007, Hou et al. 2009). The primers used are LCO1490 (5’-GGTCAACAAATCATAAAGATATTGG-3’) and HCO2198 (5’-TAAACTTCAGGGTGACCAAAAAATCA-3’) (Folmer et al. 1994). Genomic DNA extraction, amplification and sequencing procedures were performed as in Hou et al. (2007). Pairwise uncorrected sequence distances were calculated using PAUP* (Swofford 2001). The new sequence was deposited in GenBank (accession number MH880343).

## Taxonomy

### Infraorder Bogidiellida Hertzog, 1936

#### Family Bogidiellidae Hertzog, 1936

##### Genus *Bogidiella* Hertzog, 1933

###### Bogidiella pingxiangensis

Taxon classificationAnimaliaAmphipodaBogidiellidae

Hou & Li sp. n.

http://zoobank.org/7DE05148-BC5C-4CBD-8D58-B9936CDD0226

**Figure 1.**
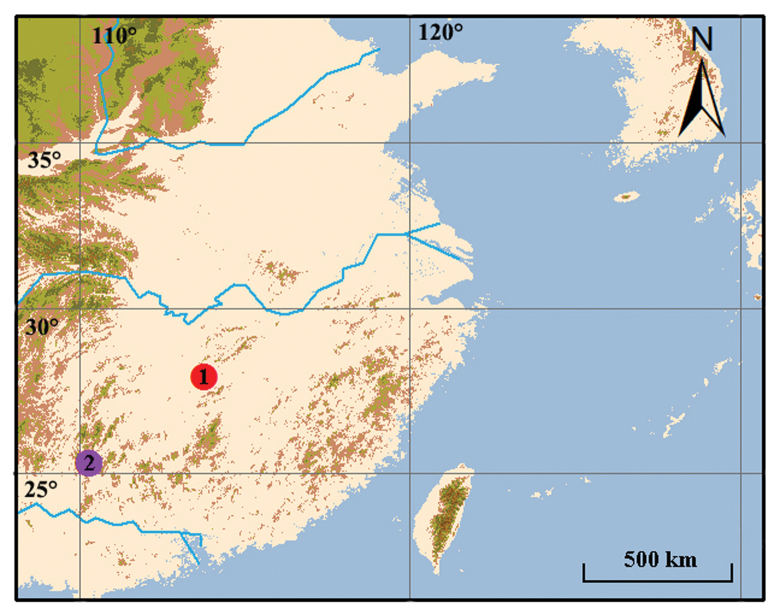
Type localities of *Bogidiella* species from China. 1 *Bogidiellapingxiangensis* sp. n. 2 *Bogidiellasinica* Karaman & Sket, 1990.

**Figure 2.**
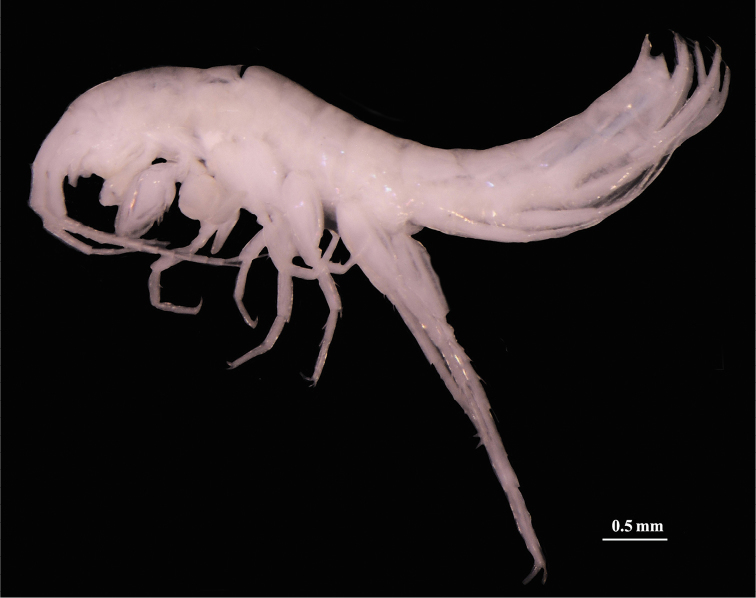
*Bogidiellapingxiangensis* sp. n., male holotype from Jiangxi, China.

**Figure 3.**
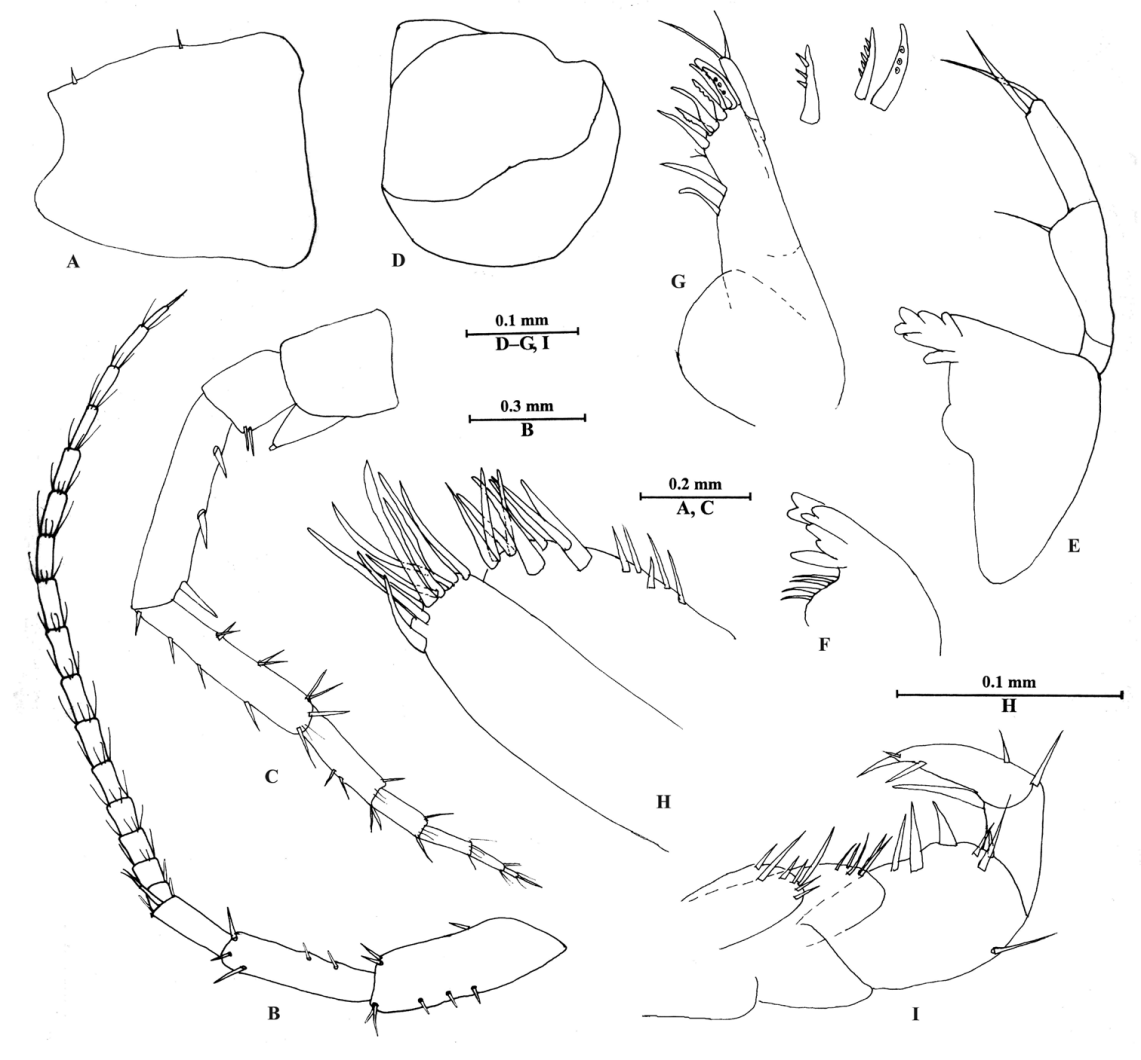
*Bogidiellapingxiangensis* sp. n. male holotype, from Jiangxi, China. **A** head **B** antenna I **C** antenna II **D** upper lip **E** left mandible **F** incisor of right mandible **G** maxilla I **H** maxilla II **I** maxilliped.

**Figure 4.**
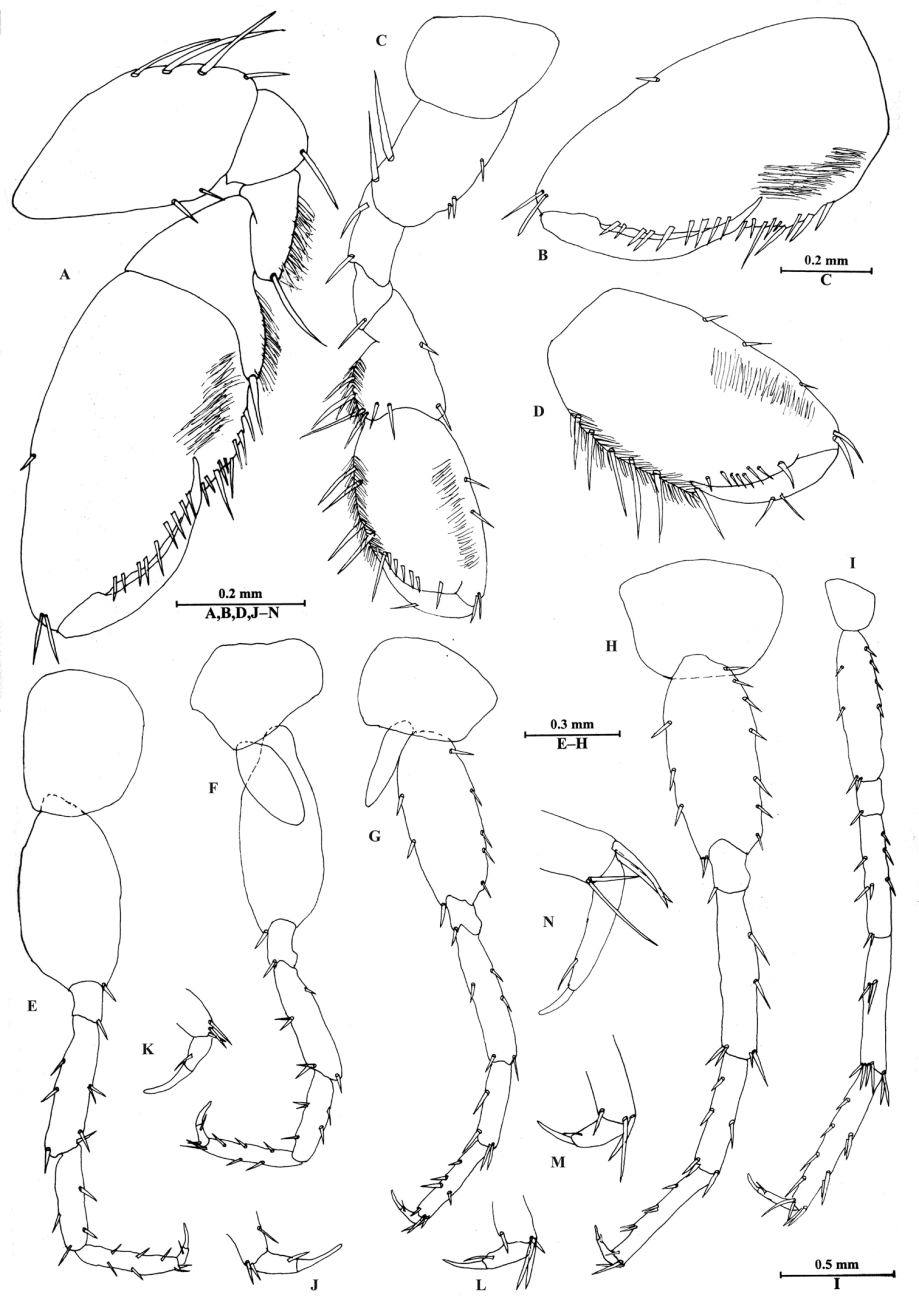
*Bogidiellapingxiangensis* sp. n., male holotype, from Jiangxi, China. **A** gnathopod I **B** propodus of gnathopod I **C** gnathopod II **D** propodus of gnathopod II **E** pereopod III **F** pereopod IV **G** pereopod V **H** pereopod VI **I** pereopod VII **J** dactylus of pereopod III **K** dactylus of pereopod IV **L** dactylus of pereopod V **M** dactylus of pereopod VI **N** dactylus of pereopod VII.

**Figure 5.**
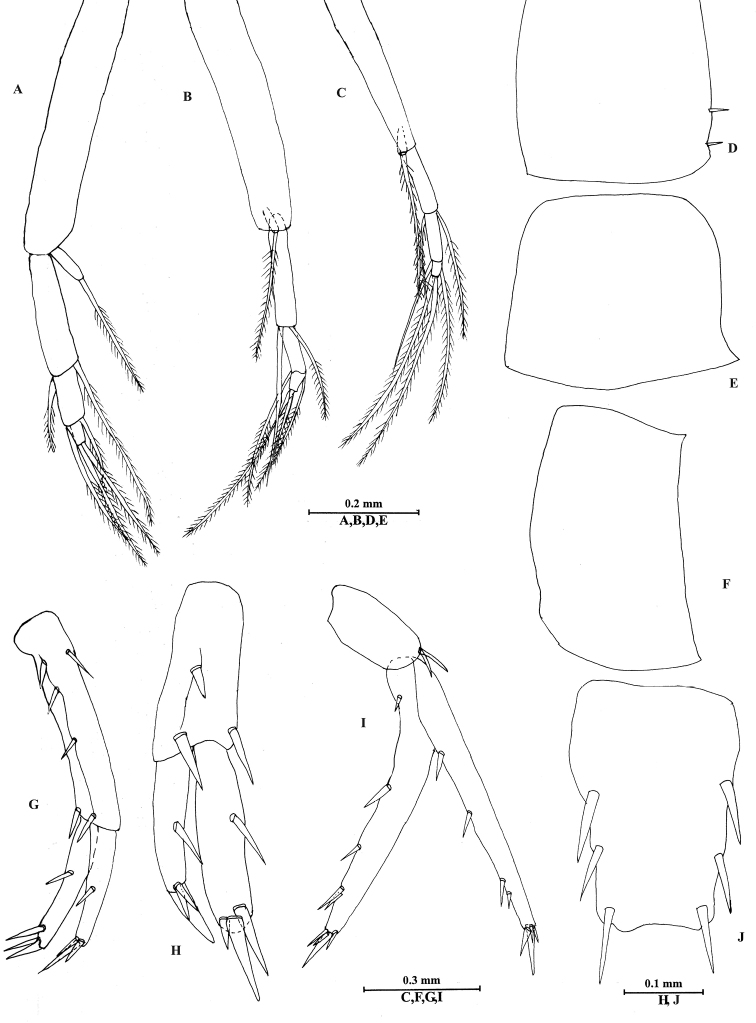
*Bogidiellapingxiangensis* sp. n., male holotype, from Jiangxi, China. **A** pleopod I **B** pleopod II **C** pleopod III **D** epimeral plate I **E** epimeral plate II **F** epimeral plate III **G** uropod I **H** uropod II **I** uropod III **J** telson.

**Figure 6.**
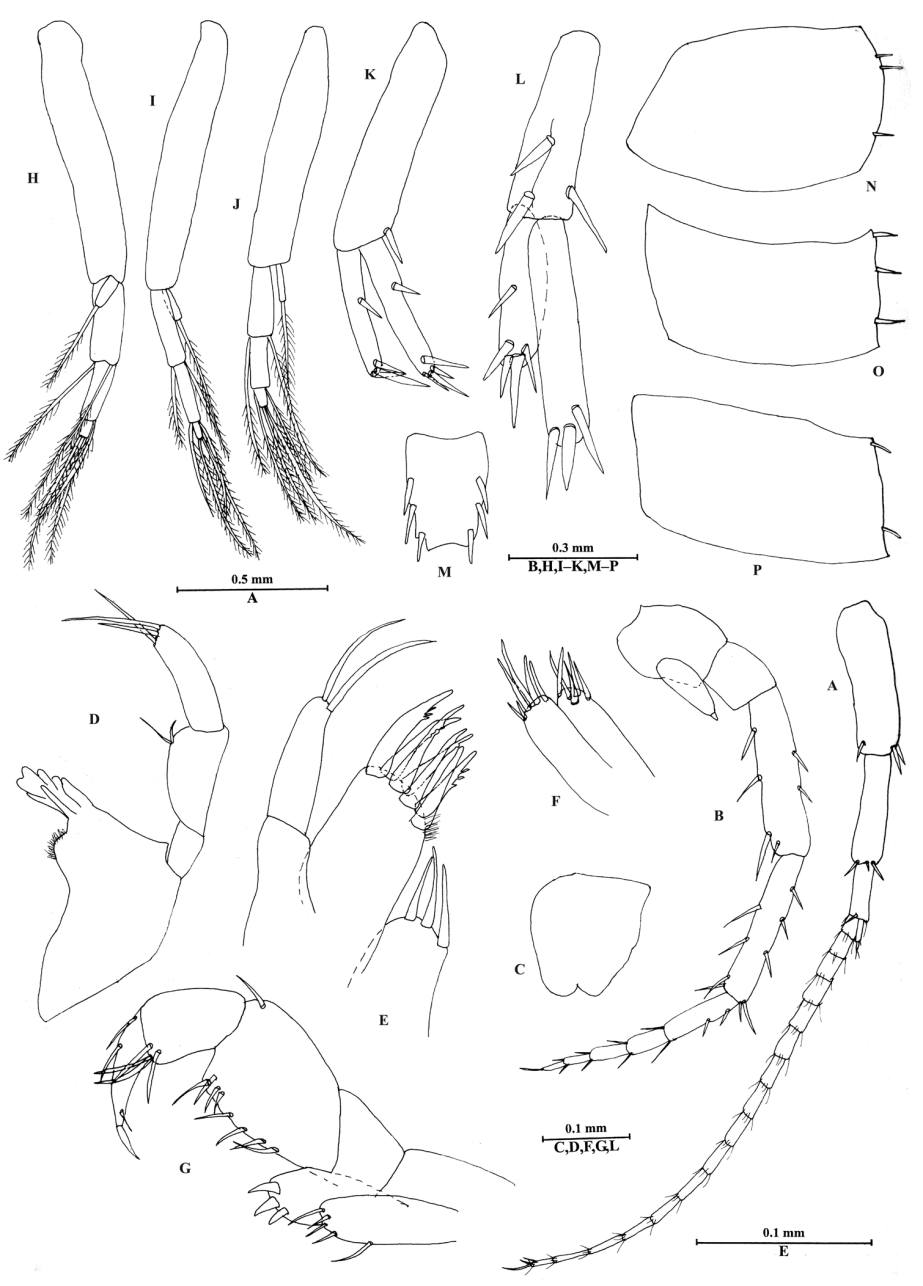
*Bogidiellapingxiangensis* sp. n., female paratype, from Jiangxi, China. **A** antenna I **B** antenna II **C** upper lip **D** left mandible **E** maxilla I **F** maxilla II **G** maxilliped **H** pleopod I **I** pleopod II **J** pleopod III **K** uropod I **L** uropod II **M** telson **N** epimeral plate I **O** epimeral plate II **P** epimeral plate III.

**Figure 7.**
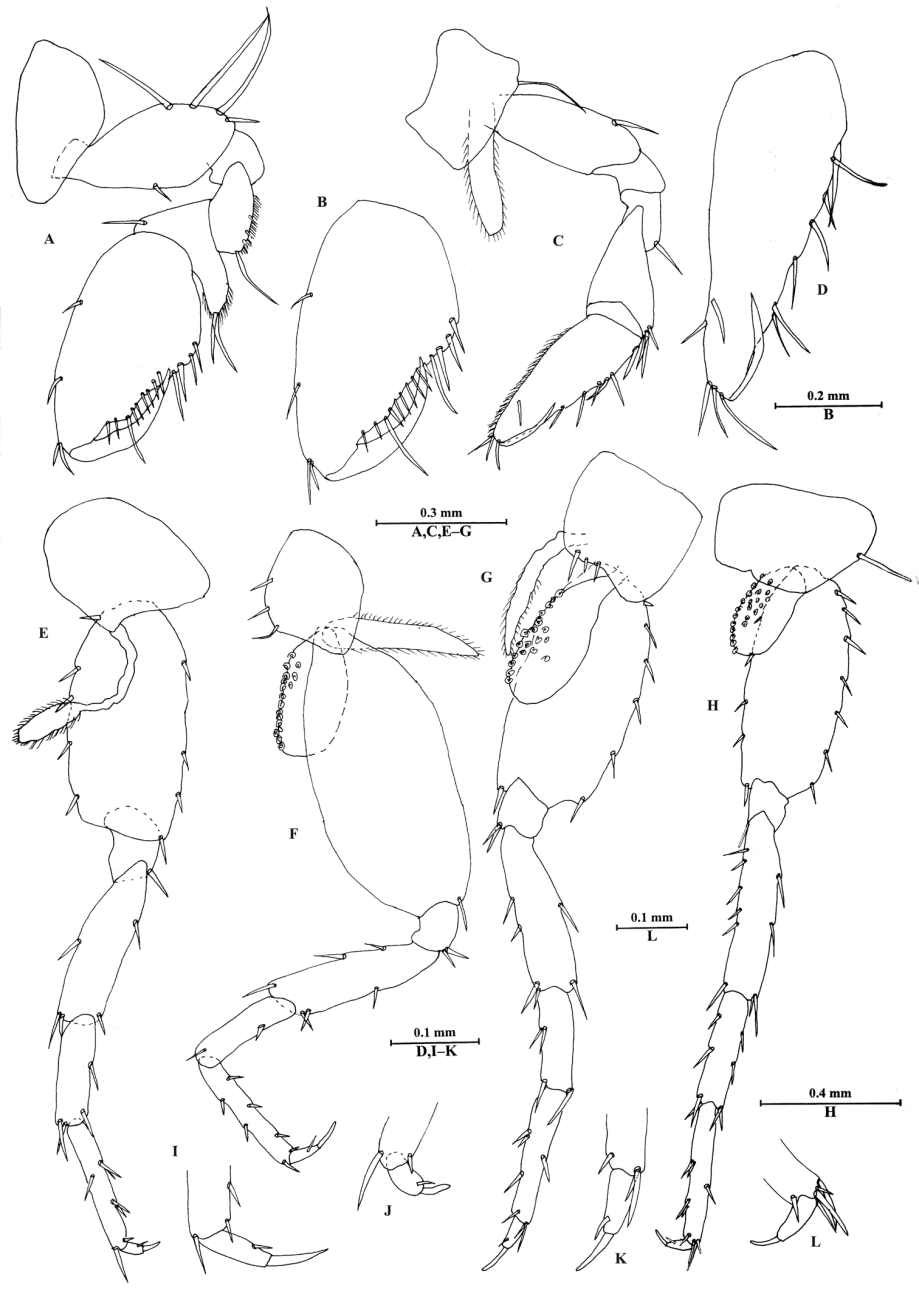
*Bogidiellapingxiangensis* sp. n., female paratype, from Jiangxi, China. **A** gnathopod I **B** propodus of gnathopod I **C** gnathopod II **D** propodus of gnathopod II **E** pereopod III **F** pereopod IV **G** pereopod V **H** pereopod VI **I** dactylus of pereopod III **J** dactylus of pereopod IV **K** dactylus of pereopod V **L** dactylus of pereopod VI.

Figs 1 , 2 , 3 , 4 , 5 , 6 , 7

####### Type species.

*Bogidiellaalbertimagni* Hertzog, 1933.

####### Material examined.

Holotype: male (IZCAS-I-A1316-1), 5.0 mm, Xiongshizilong Cave (113.76°E, 27.91°N), Changping Village, Futian Town, Shangli County, Pingxiang City, Jiangxi Province, May 9, 2013, collected by Yufa Luo and Jincheng Liu. Paratype: female (IZCAS-I-A1316-2), 4.0 mm, same data as holotype.

####### Etymology.

The specific name referes to type locality; adjective.

####### Diagnosis.

Antenna I longer than antenna II; palp of maxilla I with two apical setae; basis of gnathopod I expanded; bases of pereopods III–VI expanded, without spines and setae; coxal gills present on pereopods IV–VI; pleopod inner ramus with one segment, reduced; uropod II outer ramus shorter than inner ramus; telson 1.42 times longer than wide, apical margin with shallow U-shaped excavation, each lobe bearing one apical and two subapical stout spines.

####### Description of male holotype

(IZCAS-I-A1316-1), 5.0 mm.

*Head*. (Figure 3A): eyes absent.

Antenna I (Figure 3B): longer than antenna II, peduncle articles I–III in length ratio 1.0: 0.7: 0.4, with distal spines; flagellum with 17 articles; accessory flagellum with two articles; both primary and accessory flagellum with short distal setae.

Antenna II (Figure 3C): peduncle articles III–V in length ratio 1.0: 2.6: 2.4, peduncle article III with two distal spines, articles IV–V nearly same length, article IV with three lateral spines, article V with stiff setae along anterior and posterior margins; flagellum with six articles, each article with distal setae; calceoli absent.

Upper lip (Figure 3D): ventral margin convex.

Mandible (Figure 3E, F): asymmetrical, left mandible incisor with five teeth; lacinia mobilis small; palp composed of three articles, second article with one distal seta, third article with two distal setae. Incisor of right mandible with four teeth, lacinia mobilis bifurcate, with small teeth.

Lower lip: destroyed.

Maxilla I (Figure 3G): inner plate with two setae; outer plate with seven apical spines, including simple (naked) spines, and spines bearing one, two or multiple dentitions; palp with two articles, second article with two apical setae.

Maxilla II (Figure 3H): inner plate with five lateral setae, six apical setae, and two subapical spines; outer plate with nine setae.

Maxilliped (Figure 3I): inner plate with seven apical setae; outer plate with five setae; palp with four articles, second article with three spines on inner margin, one seta on outer margin, two setae on apical margin, third article with two spines apically, terminal article hooked, nail small.

*Pereon*. Gnathopod I (Figure 4A, B): coxal plate destroyed; basis expanded, with four spines on posterior margin, two spines on anterior margin; merus pubescent, with one long spine on posterior margin; carpus with pubescent, tapered distolateral lobe; propodus twice as long as wide, approx. 14% larger than propodus of gnathopod II, with pubescent face, palmar margin crenellated only in its proximal (angular) part, palmar margin with nine short spines, posterior margin with a row of spines extending on proximolateral margin; dactylus reaching approx. 60% length of propodus.

Gnathopod II (Figure 4C, D): slender than gnathopod I, coxal plate longer than wide, with no spines and setae; basis longer than that of gnathopod I, with three short spines on anterior margin and two long spines on posterior margin; merus short, without pubescence; carpus without tapered projection, posterior margin pubescent, with some spines on anterior margin and posterior margins; propodus 1.7 times as long as wide, subrectangular, with a row of very fine pubescent hairs on medial surface, palmar margin with a row of short spines, posterior margin with five long spines; dactylus reaching palmar corner, with two spines on posterior margin.

Pereopods III–IV (Figure 4E–F, J–K): similar to each other, coxal plate irregular, with no spines and setae; basis extremely expanded, without spines and setae, basis of pereopod III wider than those of pereopods IV–VII; merus to propodus with some spines along anterior and posterior margins; dactylus with one setae at hinge of unguis.

Pereopods V–VII (Figure 4G–I, L–N): similar in shape. Pereopod V (Figure 4G, L) coxal plate longer than wide, with no spines and seta; basis slightly dilated but linear, with two spines on anterior margin and five spines on posterior margin; merus to propodus slender, with spines on anterior and posterior margins; dactylus with one seta at hinge of unguis. Pereopod VI (Figure 4H, M) longer than pereopod V, basis wider than that of pereopod V, with four spines on anterior margin and seven spines on posterior margin; merus bare on anterior margin and with two spines on posterior margin; carpus shorter than merus and propodus, with two spines on anterior margin and one spine on posterior margin; propodus with three pairs of spines on anterior margin; dactylus with one seta at hinge of unguis. Pereopod VII (Figure 4I, N) nearly twice as long as pereopods V–VI, basis linear, with two short spines on anterior margin and four spines on posterior margin; carpus longer than merus, with a pair of spines on anterior margin; propodus with four long spines on anterior margin and two pairs of spines on posterior margin; dactylus elongate, with a seta at hinge of unguis.

Coxal gills present on pereopods IV–VI.

*Pleon*. Epimeral plates (Figure 5D–F): plate I ventrally rounded, with two setae on posterior margin; plate II posterior corner acute; plate III posterior corner blunt.

Pleopods I–III (Figure 5A–C): similar to each other, inner ramus short, with a long and plumose distal seta; outer ramus 3-articulate, each article with two long, plumose setae which are longer towards the tip of the ramus.

*Urosome*. Uropod I (Figure 5G) peduncle longer than rami, with one basofacial spine, one and four spines on inner and outer margins, respectively; inner ramus slightly longer than outer ramus, bearing one spine on inner margin; outer ramus with one spine on outer margin; both rami with three terminal spines. Uropod II (Figure 5H) peduncle longer than outer ramus but shorter than inner ramus, with one and two spines on inner and outer margins, respectively; inner ramus stronger than outer ramus, with one spine on inner margin; outer ramus with one spine on inner margin, both rami with three terminal spines. Uropod III (Figure 5I) longer than uropods I–II, peduncle approx. 1/3 the length of rami, with two spines on distal margin; inner and outer ramus rod-shaped, both with four to five marginal spines and four terminal spines.

Telson (Figure 5J): length 1.42 times as width, apical margin with shallow U-shaped excavation, each lobe bearing one apical and two lateral stout spines.

####### Description of paratype female

(IZCAS-I-A1316-2), 4.0 mm.

*Head*. Antenna I (Figure 6A): peduncle articles with distal spines, flagellum with 16 articles, accessory flagellum with two articles.

Antenna II (Figure 6B): peduncle articles IV–V with three to four spines along anterior and posterior margins; flagellum with five articles, the first article twice as long as second article.

Upper lip convex (Figure 6C).

Mandible (Figure 6D): incisor with five teeth; lacinia mobilis small; palp with three articles, the second article expanded, with two setae, the third article with three distal setae.

Maxilla I (Figure 6E): inner plate with three distal setae; outer plate with seven serrated spines; second article of palp with two apical setae.

Maxilla II (Figure 6F): inner and outer plates with several setae.

Maxilliped (Figure 6G): inner plate with four setae; outer plate with three stout spines; second article of palp expanded, with eight marginal setae, third article short, fourth article claw-shaped.

*Pereon*. Gnathopod I (Figure 7A, B): similar to that of male. Basis expanded; carpus with tapered projection; propodus 2.7 times as long as that of gnathopod II, palmar margin with a row of 13 spines.

Gnathopod II (Figure 7C, D): slender, merus and carpus without pubescence; propodus twice as long as wide, with a row of very fine pubescent hairs on anterior side; posterior margin with a row of seven spines.

Pereopods III–VI (Figure 7E–L): similar to those of male, basis expanded.

Coxal gills present on pereopods IV–VI, with little bumps.

Oostegites present on gnathopod II and pereopods III–V.

*Pleon*. Epimeral plates I–III (Figure 6N–P): plate I–III with three, three and two setae on posterior margin respectively.

Pleopods I–III (Figure 6H–J) similar to those of male, inner ramus short.

*Urosome*. Uropod I (Figure 6K): peduncle without basofacial spine; both rami with three to four terminal spines. Uropod II (Figure 6I): outer ramus distinctly shorter than inner ramus. Uropod III missing.

####### Habitat.

This species was collected from a cave, with rotten wood.

####### Remarks.

The new species is assigned to the *Bogidiella*-*skopljensis* group (group B) according to inner ramus of pleopod with one segment (Koenemann and Holsinger 1999). *Bogidiellapingxiangensis* sp. n. is similar to *Bogidiellasinica* Karaman & Sket, 1990 in having antenna I longer than antenna II; gnathopod I distinctly larger than gnathopod II; and pleopods I–III inner ramus short. *Bogidiellapingxiangensis* sp. n. differs from *B.sinica* (character states in parentheses) by gnathopod I basis expanded, rectangular (weakly expanded, elongate); pereopods III–IV bases expanded (slightly dilated but linear); telson 1.42 times longer than wide, apical margin with shallow U-shaped excavation, each lobe bearing one apical and two lateral stout spines (wider than long, with a straight distal margin bearing two disto-lateral spines which are longer than the telson itself, with three short plumose setae near each spine).

The new species is also similar to *Bogidiellaveneris* Leijs, Bloechl & Koenemann, 2011 in having antenna I longer than antenna II; second article of palp in maxilla I with two apical setae; and in the shape of gnathopods I–II. *Bogidiellapingxiangensis* sp. n. differs from *B.veneris* (character states in parentheses) by articles III–IV of maxilliped without pubescent surfaces (with pubescent surfaces); bases of pereopods III–VII expanded (linear); propodus of pereopod VII with short spine (propodus with a cluster of long, posterodistal setae); inner ramus of pleopods I–III short (inner ramus absent); telson 1.42 times longer than wide, apical margin with shallow U-shaped excavation, each lobe bearing one apical and two subapical stout spines (small, as long as wide, with straight distal margin, equipped with two spines).

We downloaded all nine COI sequences of the genus *Bogidiella* from GenBank, including six for *B.albertimagni* Hertzog, 1933, two for *B.indica* Holsinger, Reddy & Messouli, 2006, and one for *B.veneris* Leijs, Bloechl & Koenemann, 2011. Molecular analyses showed high interspecific divergences. The uncorrected pairwise distance between *Bogidiellapingxiangensis* sp. n. and *B.albertimagni*, *B.indica*, *B.veneris* is 23.5–26.8% for COI. This value is larger than COI threshold (16%) for crustacean species delimitation (Lefébure et al. 2006). Therefore, morphological and molecular data support *B.pingxiangensis* sp. n. being a new species.

## Supplementary Material

XML Treatment for Bogidiella pingxiangensis
